# Comparative Effect of Celecoxib, Diclofenac, and Ibuprofen in Controlling Postoperative Pain, Edema, and Trismus After Third Molar Extraction: A Double-Blinded Randomized Controlled Trial

**DOI:** 10.7759/cureus.53687

**Published:** 2024-02-06

**Authors:** Lojain Bassyoni

**Affiliations:** 1 Department of Oral and Maxillofacial Surgery, King Abdulaziz University faculty of Dentistry, Jeddah, SAU

**Keywords:** trismus, postoperative pain, impacted third molars, nsaids, celecoxib

## Abstract

The objective of this study was to compare celecoxib, diclofenac, and ibuprofen for managing postoperative pain, swelling, and trismus after a third molar extraction. There were 90 patients included and randomly allocated, 30 in each of the three study groups. The primary outcome of this trial was postoperative pain, and the secondary outcomes were postoperative swelling and trismus. The celecoxib and diclofenac groups showed better postoperative pain control compared to ibuprofen. Moreover, diclofenac showed better pain control compared to both celecoxib and ibuprofen within the first 72 hours postoperatively: one hour (p=0.005), six hours (p=0.001), 12 hours (p=0.044 ), 24 hours (p=0.017), 48 hours (p=0.006), and 72 hours (p=0.012 ). Regarding the secondary outcomes, there was no statistical difference in the swelling and trismus measurements during the postoperative period between the three study groups. The results of this study showed that celecoxib pain management post-third molar extraction is comparable to that of diclofenac and superior to that of ibuprofen.

## Introduction

Surgical extraction of wisdom teeth under local anesthesia is a routine procedure at the oral and maxillofacial surgery clinic. Common postoperative symptoms include pain, swelling, and limited mouth opening, which may persist for several days due to the trauma caused by the surgical intervention. These factors notably impact the patient’s quality of life, especially during the initial three days following surgery [1,2].

It is a common practice to prescribe analgesics and anti-inflammatory medications to alleviate the postoperative experience for patients. Typically, non-steroidal anti-inflammatory drugs (NSAIDs), such as ibuprofen and diclofenac potassium, are prescribed [3,4]. As non-selective NSAIDs, they inhibit the activity of both COX-1 and COX-2 enzymes. While these medications effectively reduce edema, inflammation, and postoperative pain, they also inhibit platelet aggregation and increase the risk of GI bleeding and renal function disturbance [5,6].

Conversely, selective NSAIDs offer similar benefits without the risks of GI bleeding or renal injury. However, their efficacy in managing postoperative dental pain is not firmly established. Celecoxib has demonstrated a comparable analgesic effect to ibuprofen, with a lower incidence of GI side effects even with high doses [7,8].

In this prospective randomized double-blinded clinical trial, our objective is to compare the effectiveness of the three aforementioned NSAIDs in controlling pain, trismus, and edema following extraction of impacted lower third molars.

## Materials and methods

This was a randomized, double-blinded, single-center clinical trial conducted on patients requiring the extraction of impacted lower third molars. The participants were recruited from the oral and maxillofacial surgery specialty clinics at King Abdul-Aziz University Dental Hospital in Jeddah, Saudi Arabia. Informed consent was obtained from all patients participating in the trial. The recruitment process extended from November 1, 2022, until February 28, 2023. The ethical approval for this study was obtained from the Biomedical Ethics Committee at King Abdul-Aziz University in Saudi Arabia, Faculty of Dentistry (approval number: 323-11-21).

Inclusion and exclusion criteria

Patients aged between 18 and 40 years with no systemic disease (ASA I status), no allergies to any of the study drugs, and no local or systemic infection were included in the study. Asymptomatic mesioangular impactions were also included.

All patients with pericoronitis, temporomandibular joint dysfunction, myofascial pain, pathology, or infection related to their impacted wisdom teeth were not included in the study population. Only one impacted lower third molar was extracted at the time of surgery.

The patients were randomly allocated to one of the three different postoperative NSAID protocols (celecoxib, diclofenac potassium, or ibuprofen) using a simple randomization technique. This was done using a computer-generated random number sequence. The sequence was used to assign patients to the treatment groups in a 1:1:1 ratio as they were recruited into the study. The treatment protocols were (i) celecoxib 100 mg po Q12h x 3 days; (ii) diclofenac 50 mg po Q8h x 3 days; and (iii) ibuprofen 400 mg po Q6h x 3 days. In all three protocols, paracetamol 1 g po q6h PRN (when necessary) was prescribed as a rescue medication. In addition to demographic data, the following parameters were measured and collected during follow-up visits on days 2, 4, and 7.

Pain

**Figure 1:**
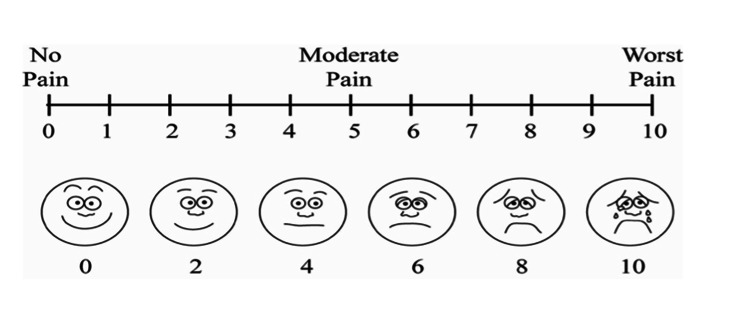
Visual analog scale (VAS)

The patients' pain levels after the procedure were assessed at predetermined time points using a 10-cm visual analogue scale (VAS; Figure 1), ranging from 0 (indicating no pain) to 10 (indicating maximum pain). Patients were requested to record their pain levels at one, six, 12, 24, 48, and 72 hours after surgery. Additionally, the number of rescue medication pills used by the patients was documented at the end of each day. Furthermore, pain assessments were conducted by two calibrated examiners during follow-up visits on postoperative days 2, 4, and 7, following the same method.

Edema

Edema measurements were performed by two examiners to evaluate baseline and postoperative edema on the surgical side. The extent of edema was assessed using three linear measurements based on the Gabka-Matsumura technique [9]. The first measurement (S1) involved the distances between the angle of the mandible and the corner of the eye, while the second measurement (S2) extended from the tragus of the ear to the corner of the mouth. The third measurement was from tragus to pogonion (S3). All measurements were taken with the patients in an upright sitting position and obtained using dental floss and a ruler. These measurements were recorded on postoperative days 2, 4, and 7.

The two examiners were calibrated. A training workshop was held, describing the standardized application of the Gabka-Matsumura technique for measuring edema and the consistent use of the caliper to assess trismus. Interexaminer reliability was assessed, as both examiners independently measured edema and trismus on a sample of 10 individuals not included in the study. The measurements of both examiners were compared, and the intra-class correlation coefficient was calculated.

Trismus

To measure trismus, the distance between the upper and lower incisors was assessed during maximum opening using a caliper. These measurements were obtained by the same two examiners on postoperative days 2, 4, and 7.

Procedure

The patients were provided with an explanation of the procedure and the nature of the study. Upon their approval to participate and after signing the consent form, demographic data, medical history, and baseline measurements for trismus and swelling were collected.

All procedures were carried out by four calibrated senior-level residents under the supervision of the same consultant. A training session was held for the four senior residents who performed the procedures, focusing on the standardized technique for third molar extraction. A calibration session was also held, where each resident performed two extractions under supervision.

The procedures were performed under local anesthesia (mepivacaine 2% with 1:100,000 epinephrine), and two carpules of the local anesthetic were used for each patient. No other medications were administered during the procedure. A standardized technique was followed for the extraction of one impacted mandibular third molar in each participant. The procedure involved the following steps: incision, mucoperiosteal flap reflection, bone removal, tooth sectioning, tooth delivery, bone filing, irrigation using normal saline, and suturing. No other teeth were extracted during the procedure.

The residents who performed the procedure and the two examiners who conducted the postoperative measurements were blinded to the medication given to the patient. The medications were coded as A, B, and C; they were dispensed to the patients in opaque bottles with instructions on how to use them. Their decoding was carried out only after the conclusion of the statistical analysis.

Statistical analysis

Data were analyzed using RStudio 2023.06.0 (R version 4.3.0, R Foundation for Statistical Computing, Vienna, Austria), and the level of significance was set at p<0.05. Descriptive statistics were performed to assess the mean and standard deviation. The normality of the data was assessed using the Shapiro-Wilk test. Inferential statistics to find out the difference within and between the groups were done using an independent t-test and a one-way ANOVA with the Mann-Whitney U test and the Kruskal-Wallis test as non-parametric alternatives when the normality assumption was violated. The chi-square test was used to measure the association between categorical variables.

## Results

This study compared the effectiveness of celecoxib 100 mg, ibuprofen 400 mg, and diclofenac 50 mg in 90 participants. The enrolled subjects (52% females) were randomly allocated into the three treatment groups, with 30 patients in each. The mean age of the patients enrolled in this study was 25.87±5.64, 25.3±6.56, and 23.27±3.89 in celecoxib, diclofenac, and ibuprofen, respectively. The procedure duration in minutes between the groups was the celecoxib group, 25.97±7.24; the diclofenac group, 26.27±11.48; and the ibuprofen group, 27.17±12.31. There was no statistical significance in gender, age, or treatment duration between the three treatment groups (p>0.05).

**Figure 2:**
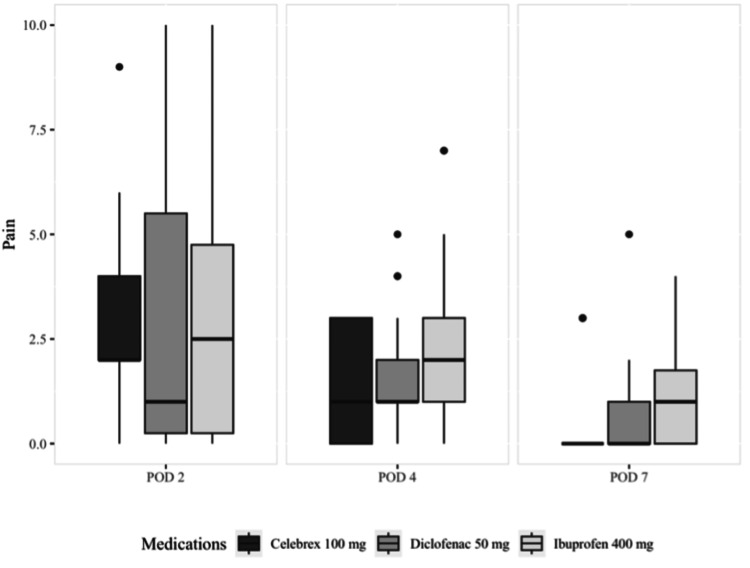
Median and quartiles of pain POD: postoperative day

Table 1 and Figure 2 show the mean, standard deviation, and median (quartiles) of pain. No significant difference was observed on the second postoperative day (p>0.05). However, the pain intensity was significantly different between the groups on the fourth and seventh postoperative days, with the ibuprofen group having the highest pain score (p<0.05).

**Table 1:** Comparison of postoperative pain intensity reported by the examiners across the groups* Multiple comparisons testing was done, and significant differences were observed at POD, between celebrex and ibuprofen (p=0.021), between diclofenac and ibuprofen (p=0.009), and between celebrex and ibuprofen (p=0.031) * Kruskal-Wallis test was used, ** significant at p<0.05 POD: postoperative day

Time	Celecoxib 100 mg (mean±SD)	Diclofenac 50 mg (mean±SD)	Ibuprofen 400 mg (mean±SD)	p-value
POD 2	2.97±2.2	2.7±2.95	2.9±2.8	0.644
POD 4	1.47±1.31	1.43±1.25	2.7±1.93	0.007**
POD 7	0.5±1.14	0.57±1.04	1.1±1.35	0.027**

**Figure 3:**
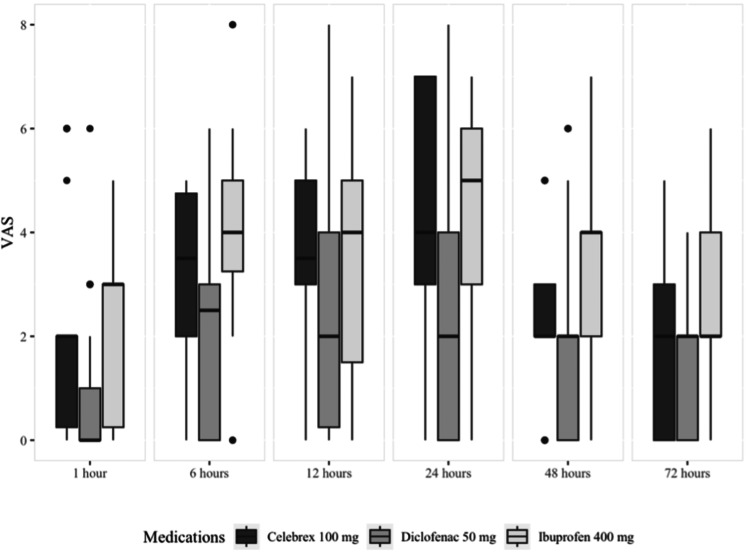
Median and quartiles of VAS score VAS: visual analogue scale

According to the VAS scores reported by the patients demonstrated in Table 2 and Figure 3, the pain peaked at 24 hours postoperatively in the celecoxib and ibuprofen groups and at 12 hours in the diclofenac group. However, the mean VAS scores were significantly lower in the diclofenac group at one hour, six hours, 12 hours, 24 hours, 48 hours, and 72 hours postoperatively when compared to the other two medication groups.

**Table 2:** Comparison of postoperative pain intensity reported by the patients across the groups* Multiple comparison testing was done and the following pairs were statistically significant. One hour: celebrex vs. diclofenac (p=0.021) and diclofenac vs. ibuprofen (p=0.007). Six hours: celebrex vs. diclofenac (p=0.033) and celebrex vs. ibuprofen (p=0.046). 24 hours: celebrex vs. diclofenac (p=0.031) and diclofenac vs. ibuprofen (p=0.026). 48 hours: celebrex vs. ibuprofen (p=0.029) and diclofenac vs. ibuprofen (p=0.016). 72 hours: celebrex vs. ibuprofen (p=0.030) and diclofenac vs. ibuprofen (p=0.018). The test used for the pairwise comparisons is Wilcoxon rank-sum test * Kruskal-Wallis test was used, ** significant at p<0.05 VAS: visual analogue scale

Time	Celecoxib 100 mg (mean±SD)	Diclofenac 50 mg (mean±SD)	Ibuprofen 400 mg (mean±SD)	p-value
1 hour	2.07±2.08	0.93±1.41	2.4±1.87	0.005**
6 hours	3.13±1.68	2.1±1.77	4.17±1.78	0.001**
12 hours	3.53±1.74	2.7±2.42	3.8±2.3	0.044**
24 hours	4.03±2.53	2.43±2.79	4.17±2.28	0.017**
48 hours	2.23±1.36	1.87±1.89	3.37±2.14	0.006**
72 hours	1.63±1.43	1.53±1.36	2.67±1.49	0.012**

**Figure 4:**
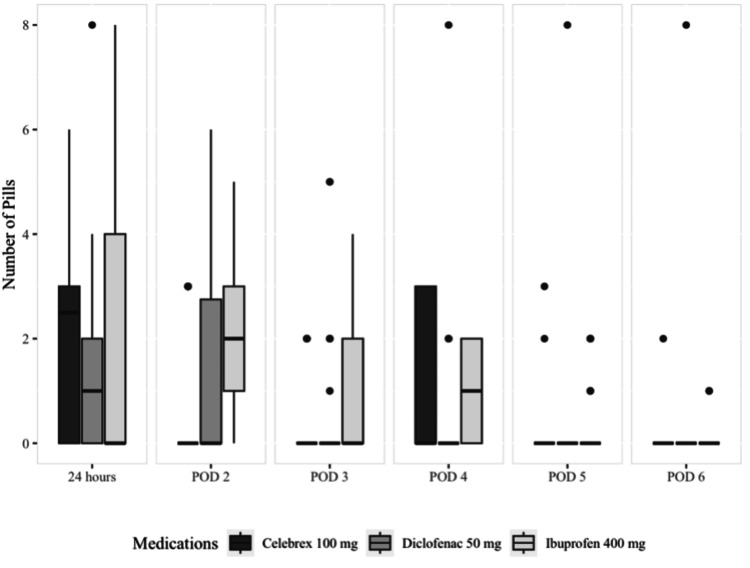
Median and quartiles of the number of pills used for rescue medication POD: postoperative day

​​​The number of pills of rescue medication paracetamol utilized by the groups was statistically significant on postoperative days 2 (p=0.007), 4 (p=0.020), and 5 (p=0.045). Patients in the celecoxib and diclofenac groups consumed the least number of rescue medication pills (Figure 4).

**Figure 5:**
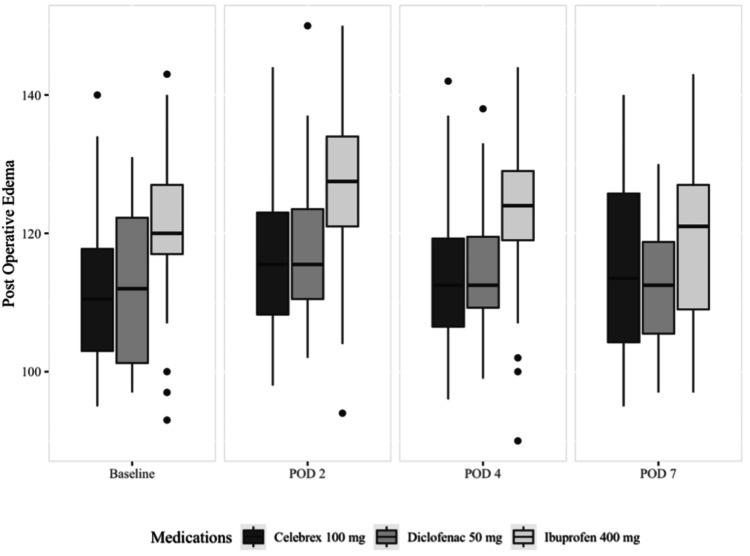
Median and quartiles of edema POD: postoperative day

Table 3 and Figure 5 display the mean, standard deviation, median (quartiles), and significance of postoperative edema. There wasn’t a statistically significant difference between the groups at any time point (p>0.05).

**Table 3:** Comparison of postoperative edema among the celecoxib, diclofenac, and ibuprofen groups* * one-way ANOVA test was used, ** non-normality of distribution in groups, Kruskal-Wallis test (decisions regarding normality were made based on the Shapiro-Wilk test) POD: postoperative day

Medication	Baseline (mean±SD)	POD 2 (mean±SD)	POD 4 (mean±SD)	POD 7 (mean±SD)	p-value
Celecoxib 100 mg	111.37±11.31	116.87±11.96	113.57±11.62	114.7±12.62	0.347
Diclofenac 50 mg	112±10.91	117.77±11.42	114.73±10.28	112.43±9.1	0.178 NP**
Ibuprofen 400 mg	119.57±11.54	125.93±12.58	121.87±11.85	118.87±12.13	0.103

**Figure 6:**
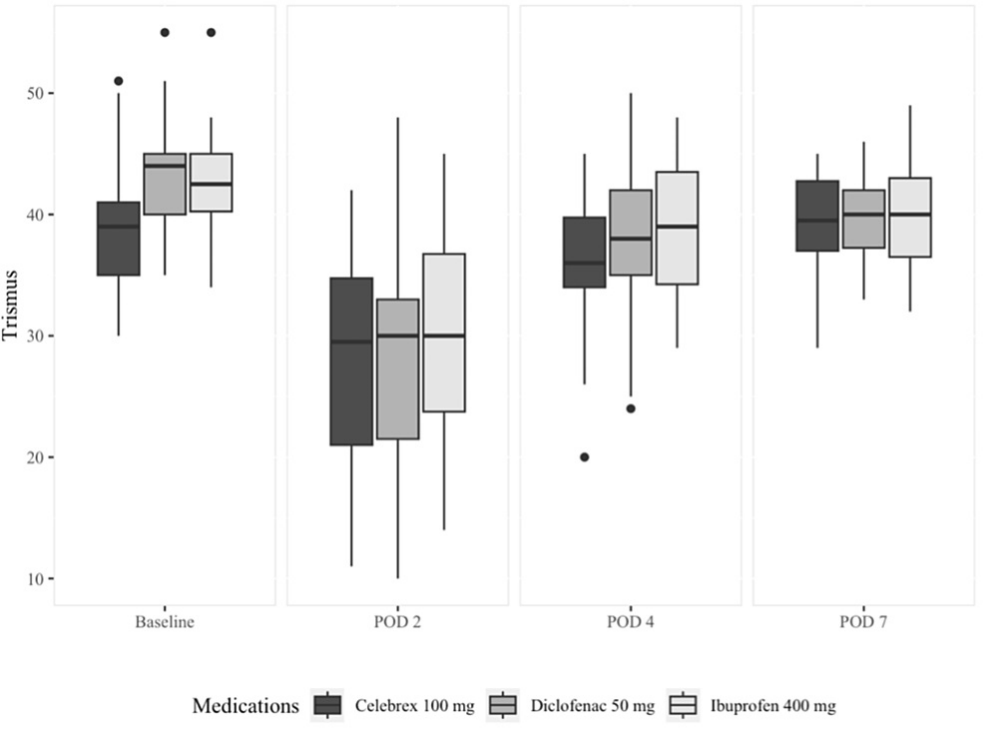
Median and quartiles of trismus measurements POD: postoperative day

Table 4 and Figure 6 display the mean, standard deviation, median (quartiles), and significance of trismus. As viewed, there wasn’t a statistically significant difference between the three study groups at any time point except at baseline, which is not clinically relevant (p>0.05). However, the reduction in mouth opening from baseline to postoperative day 2 is the lowest in the celecoxib group, followed by the ibuprofen group, and lastly, the diclofenac group. Additionally, the celecoxib group was the quickest to go back to their baseline mouth opening by postoperative day 7.

**Table 4:** Comparison of trismus progression over postoperative day 2 among celecoxib, diclofenac, and ibuprofen groups* * a one-way ANOVA test was used. Multiple comparison results indicated that the statistically significant difference in trismus in the pairs was observed between celebrex and diclofenac (p=0.002) POD: postoperative day

Time	Celecoxib 100 mg (mean±SD)	Diclofenac 50 mg (mean±SD)	Ibuprofen 400 mg (mean±SD)	p-value
Baseline	39±4.88	43.2±4.6	42.63±4.26	0.001
POD 2	28.1±8.68	27.3±9.2	30.8±8.78	0.284
POD 4	35.93±5.37	38.03±6.19	38.63±5.47	0.161
POD 7	39.4±4.01	39.83±3.62	40.47±4.49	0.593

## Discussion

In this study, we compared the effectiveness of celecoxib, diclofenac, and ibuprofen in controlling pain, edema, and trismus after lower third molar extraction. Celecoxib and diclofenac were more effective in pain management than ibuprofen. There was, however, no significant difference in postoperative swelling and trismus among the three groups.

Postoperative swelling following third molar surgery typically peaks 48 hours after the procedure. Various methods for measuring postoperative edema are documented in the literature, including photography, ultrasound measurements, facial arch assessment, and measurements between facial anatomical landmarks [10-15]. In this study, we employed an objective craniometric method to measure postoperative edema, as detailed in the methodology [9].

The research has consistently demonstrated the effectiveness of NSAIDs in managing postoperative pain and discomfort following third molar surgery [16,17]. The findings of our study indicate that the efficacy of celecoxib 100 mg in controlling postoperative pain after third molar extraction is comparable to diclofenac 50 mg and superior to ibuprofen 400 mg. Additionally, the data revealed that patients in the diclofenac and celecoxib groups consumed significantly less (paracetamol) compared to the ibuprofen group, suggesting superior performance of diclofenac and celecoxib based on our findings.

These results are consistent with a study published by Gazal et al., which compared ibuprofen and paracetamol to diclofenac potassium and found that diclofenac was more effective in reducing pain after tooth extraction and deep cavity preparation [18]. However, it’s worth mentioning that they only included patients who underwent elective simple tooth extraction.

Furthermore, Akinbade et al. conducted a comparative analysis of celecoxib, ibuprofen, and tramadol in managing postoperative pain after third molar surgery, concluding that celecoxib was the most effective analgesic, followed by ibuprofen, with tramadol being the least effective [19]. Similarly, a study by Isola et al. comparing celecoxib and ibuprofen found that the celecoxib group exhibited a significant reduction in postoperative pain compared to the ibuprofen and placebo groups [20].

These consistent findings across multiple studies support the conclusion that celecoxib and diclofenac are more effective in managing postoperative pain after third molar surgery compared to ibuprofen. The study by Esteller-Martínez et al. that found no statistical differences in the analgesic effect of diclofenac sodium and ibuprofen after third molar surgery presents a contrasting viewpoint to our results [21]. These conflicting findings highlight the variability in research outcomes within the field of postoperative pain management, necessitating further investigation and consideration of multiple sources of evidence.

In our study, the secondary outcomes focused on postoperative edema and trismus, which are both consequences of the inflammatory response triggered by surgical trauma. Postoperative edema is a result of the inflammatory process initiated by the surgical intervention. It starts with the release of arachidonic acid from the phospholipase membrane of the cells at the surgical site, leading to the production of prostaglandins and cyclooxygenases.

Our findings indicated that the degree of postoperative edema was comparable across the three treatment groups, with no significant difference observed at any observation time point. These results align with the findings of Isola et al. [21]; however, Jha et al. demonstrated evidence that a higher dose of celecoxib (200 mg) had a superior effect in controlling postoperative swelling and pain in third molar surgery when compared to diclofenac [22].

These observations are consistent with the inflammatory response timeline usually observed after surgical trauma, as the peak inflammatory response usually occurs 24 hours after surgery, which could be reflected as edema, trismus, and pain. In our study, we observed that the maximum limitation in mouth opening in all three groups was noted on postoperative day 2. This finding is supported by other studies [23,24], reinforcing the validity of our findings in this aspect.

The varying perspectives from these studies underscore the complexity of pain management and the multifactorial nature of postoperative outcomes following third molar surgery. It is essential to consider these contrasting findings when interpreting the efficacy of different medications in managing postoperative pain and associated outcomes.

In the evaluation of trismus, which involved comparing the maximum interincisal opening at baseline and follow-up visits, our study did not reveal any significant differences in the development and resolution of trismus postoperatively among the three medication groups. This finding aligns with the work of Isola et al., as they also found no significant difference between their treatment groups in maximum mouth opening at any observation time points when comparing celecoxib to ibuprofen. The only significant difference they were able to demonstrate was in the comparison of baseline values, which is not clinically significant [20].

The study enrolled patients who underwent surgical extraction of lower wisdom teeth under local anesthesia. To ensure standardization, all procedures were supervised by the same oral and maxillofacial surgeon and performed by four calibrated senior oral and maxillofacial surgery residents. Additionally, all patients were prescribed paracetamol as a rescue pain medication. Paracetamol has a very weak COX inhibition at the periphery [25]; therefore, the consumption of this medication shouldn’t impact the study outcomes.

It's important to note that, to our knowledge, there haven't been other studies in the literature that directly compare the effects of diclofenac, celecoxib, and ibuprofen on pain, swelling, and trismus after third molar surgery. Therefore, a direct comparison of our results to previous published work is not possible.

In terms of limitations of our work, the study was conducted at a single center, and the patients included may not be fully representative of the general population undergoing third molar extraction. This could limit the generalizability of the findings to a more diverse patient population. Different patient populations, surgical techniques, and postoperative care protocols may yield different results. Therefore, larger multi-center studies are warranted to confirm the findings.

## Conclusions

The primary outcome of our study, which is focused on postoperative pain, was found to be significantly less severe in the diclofenac group, followed by the celecoxib group, compared to the ibuprofen group. Given the superior safety profile and tolerability of celecoxib, we suggest that celecoxib could be considered an option in third molar surgeries. However, this recommendation should be applied more carefully to patients with cardiac diseases. These findings underscore the need for continued research and exploration in the field of postoperative pain management, especially in the context of third molar surgery.
